# Simplified detection of a point mutation in *C. elegans* using tetra-primer ARMS-PCR

**DOI:** 10.17912/micropub.biology.000078

**Published:** 2018-12-09

**Authors:** Matthew T. Sullenberger, Eleanor M. Maine

**Affiliations:** 1 Department of Biology, Syracuse University, Syracuse, NY 13244 USA

**Figure 1.**
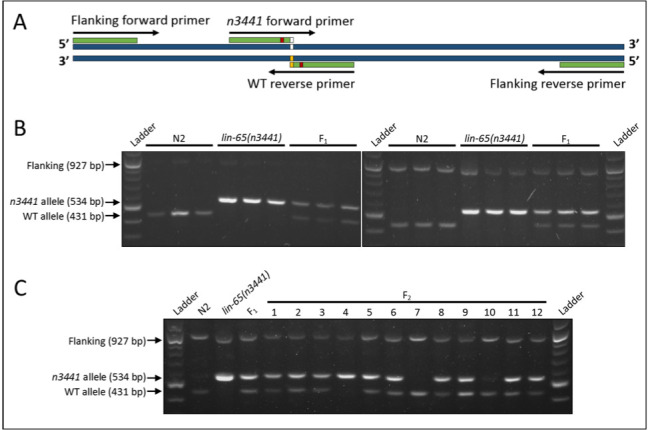
(A) Schematic of tetra-primer ARMS-PCR with *lin-65(n3441)*SNP indicated in white, WT SNP in yellow, and mismatches in red. (B) Three replicates of single-worm PCR products of *C. elegans*gene *lin-65*in N2 strain, a strain homozygous for the *lin-65(n3441)*SNP allele, and heterozygous (*n3441*/WT) F_1_progeny under standard PCR conditions (left) and conditions optimized for amplification of the SNP flanking sequence (right). (C) PCR products of parental, F_­1_, and F_2_individuals with flanking sequence amplification.

## Description

Single nucleotide polymorphisms (SNPs) can be difficult to detect using traditional PCR and gel electrophoresis, especially when no enzyme restriction sites overlap the SNP. Although alleles can be identified through amplification with flanking primers and sequencing, this adds time and cost, and sequencing is typically outsourced. Tetra-primer Amplification-Refractory MutationSystem (ARMS)-PCR allows SNPs to be distinguished through a 3’ primer mismatch at the SNP site for one allele, and an additional mismatch 1 – 3 bases upstream from the 3’ end (Figure 1A), limiting amplification to only the allele with a single mismatch (Ye*et al.*2001). Each internal primer (one forward and one reverse) is allele-specific, while external primers are different distances from the SNP, producing allele-specific fragments of different sizes in a single PCR reaction that can be distinguished by gel electrophoresis. This is useful for single-worm PCR where separate reactions may not be feasible, such as selection of individuals from a segregating population. Here, we designed primers and optimized PCR conditions to allow clear genotyping of *lin-65*(*n3441*), a G->A point mutation, without the need for sequencing. Additionally, we show that minor changes to reaction conditions allow for amplification of the larger flanking fragment (Figure 1B) which can then be gel purified and sequenced. While this may not be necessary for routine genotyping, sequencing the flanking fragment provides confirmation and higher confidence in SNP identification. To demonstrate the accuracy of our protocol, N2 and *lin-65(n3441)* adult hermaphrodites, a heterozygous F_1_hermaphrodite, and twelve segregating F­_2_progeny were genotyped by PCR using conditions optimized for amplification of the flanking fragment (Figure 1C). Flanking fragments were then gel purified and sequenced. Results showed the presence of the G allele (WT) in N2, F­_2_-7, and F_2_-10, the A allele (*n3441*) in *lin-65(n3441)* and F_2_-4, and heterozygous G/A in F_1 _and the remaining F_2_samples, confirming the genotypes indicated by PCR. Interestingly, sequencing showed a higher proportion of the G allele in all nine heterozygous samples and the heterozygous control. Those samples, as well as the homozygous *n3441* sample and control, were also heterozygous G/A at the -2 base, showing a higher proportion of the A allele; this change corresponds to the introduced G->A mismatch in the *n3441*-specific internal primer. These two observations suggest the possibility of low level overlapping PCR during amplification, incorporating the primer sequence into a portion of PCR product. Additionally, the higher proportion of the G allele at the SNP site indicates amplification bias of the *n3441-*specific primer over the flanking forward primer in the presence of the *n3441* allele, leaving the two flanking primers with mostly the WT allele for amplification in heterozygotes. This idea is supported by the visibly brighter *n3441* bands in all samples where the allele is present.

The current primer sets were chosen following the guidelines from Liu *et al.* and optimized to give the highest chance of success, though alternative primers were not tested. To overcome bias of *n3441* amplification in the larger flanking fragment, an improvement in amplification efficiency of the WT allele could be achieved by redesigning the WT-specific internal primer to be longer. Different mismatches could also be tested from the one chosen here, and at different positions relative to the SNP (-1, -3, or -4). However, we do not feel that the WT-specific primer is amplifying the WT band inefficiently, rather the *n3441*-specific primer amplifies exceptionally well; only 0.33 µl PCR product was loaded into each lane to avoid smearing due to overloading. An alternative to improving WT amplification is to slightly reduce efficiency of *n3441* amplification. This could be accomplished by following the suggestions outlined above and applying them to the *n3441*-specific primer; a slightly less efficient primer could then be selected. While tetra-primer ARMS-PCR is largely limited by the SNP site, as adjacent upstream and downstream sequence must be amenable to primer design, we have demonstrated here that certain SNPs may be worth pursuing with minimal or no changes to standard PCR protocol.

**Detailed Protocol**

Primers were developed using Multiple Primer Analyzer (Thermo Fisher Scientific). Internal allele-specific primers were designed following steps outlined by Liu *et al.* (2012). The wild-type (WT)-specific primer set included a 24-nt forward primer beginning at 412 bases upstream from the SNP, and a 19-nt reverse primer with the WT SNP (G) as its terminal (3’) base. A GC mismatch was introduced into the primer at the -2 base from the SNP site. The *n3441*-specific primer set consisted of a 20-nt reverse primer beginning 514 bases downstream from the SNP, and a 20-nt forward primer with the *n3441* SNP (A) as its terminal base. A TG mismatch was introduced into the primer at the -2 base from the SNP site. The WT and *n3441* primer sets were tested individually using single worm PCR across a temperature gradient to determine appropriate annealing temperature. Both primer sets were then combined into a single 25 µl PCR reaction with each primer at 0.2 µM, dNTP (New England BioLabs® Inc., Ipswich, MA) at 0.2 mM, 1x EconoTaq Buffer with Mg (Lucigen Corp., Middleton, WI), and 0.1 µl Taq Polymerase per reaction (http://bio-protocol.org/e-136). Samples were denatured at 94^o^C for 3 minutes, followed by 30 cycles of denaturation (94^o^C for 30 seconds), annealing (61^o^C for 30 seconds), and extension (72^o^C for 10 seconds), ending with a final extension of 72^o^C for 10 minutes. To improve amplification of the fragment flanking the SNP, external primer concentrations were increased to 0.4 µM and dNTP raised to 0.3 mM, with a 50 second extension time during the 30 cycles. All other conditions remained the same. Amplification of the WT allele, *n3441* allele, and flanking fragment generated products of 431, 534, and 927 bp, respectively.

To develop a test population, N2 males were mated with a single *lin-65*(*n3441*) hermaphrodite to produce heterozygous cross-progeny (F­­­_1_). A single F_1_hermaphrodite was isolated and allowed to produce self-progeny (F_2_). After two days of egg laying, the F_1_hermaphrodite was moved to a new plate for later genotyping.Once F_2_progeny reached adult stage, single N2 and *lin-65(n3441)* adult hermaphrodites, the F­_1 _parent, and twelve F­_2 _progeny were PCR amplified under conditions for flanking fragment amplification. 84–100 ng of PCR product per sample were loaded onto a 1% agarose gel and separated by electrophoresis at 100 V for 1.5 hours. Using 5.1 – 6.0 µg of DNA from the same PCR reaction, samples were run through a new gel and the flanking fragments purified using Monarch® DNA Gel Extraction Kit (New England BioLabs® Inc., Ipswich, MA). Purified fragments were sent to GENEWIZ (South Plainfield, NJ) for Sanger sequencing. Trace files confirmed a single peak corresponding to the allele in samples identified as homozygous by PCR. In all samples identified as heterozygous, peaks corresponding to the G and A alleles were both observed, with G peaks being noticeably higher.

## Reagents

Strains: N2 (wild-type, var. Bristol); MT13232 [*lin-65(n3441)*]. Both strains are available at the CGC.

Primer sequences: flanking forward: 5’-attcagatttccgggagaaaaatc-3’; flanking reverse: 5’-tttggtagacgtgtcgaatc-3’; WT reverse: 5’-ggcggtggaattgcttgcc-3’; *n3441*forward: 5’-cttccggcaacagtcaggta-3’
